# Supplementary material for: Single cell profiling of capillary blood enables out of clinic human immunity studies
Source: Sci Rep. 2020 Nov 25;10:20540. doi: 10.1038/s41598-020-77073-3 (PMC7688970; doi:10.1038/s41598-020-77073-3)
Supplement: Supplementary file 1 — Supplementary Information 1. [file 41598_2020_77073_MOESM1_ESM.docx]

**Supplementary Figures:**

**
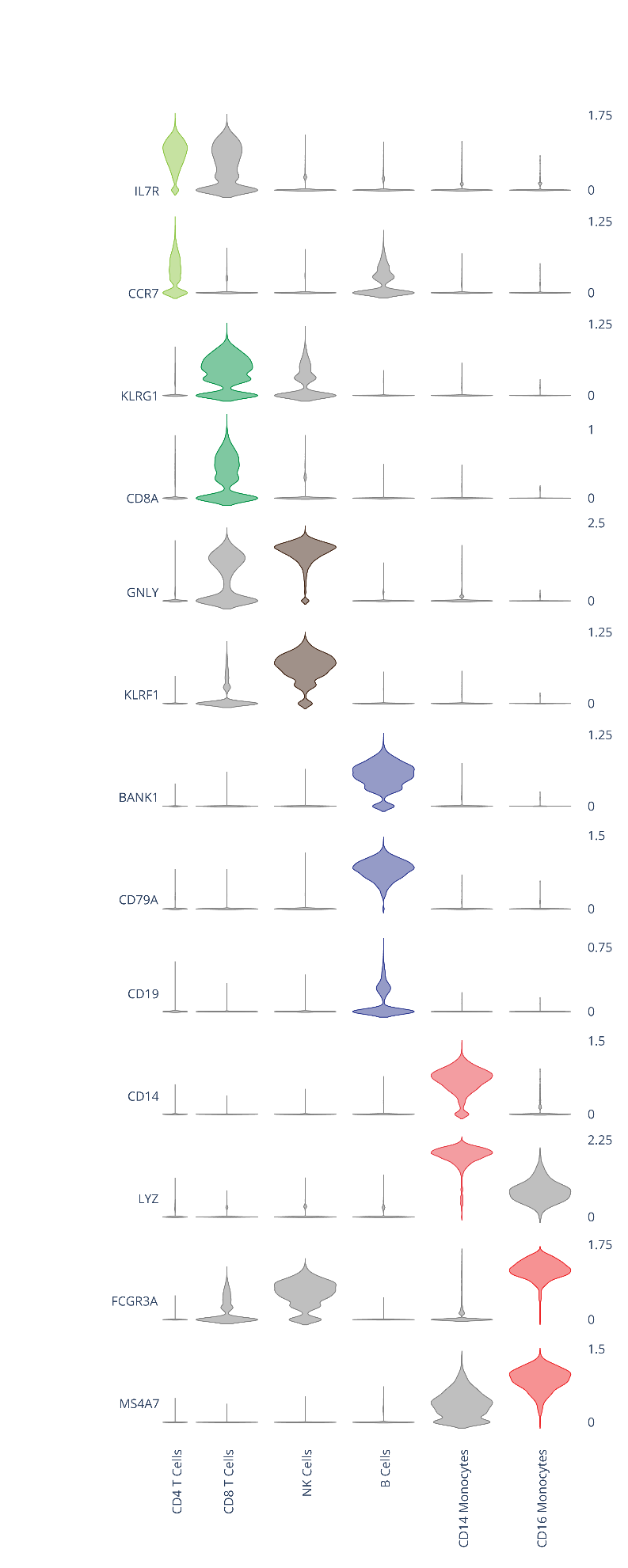
**

**Supplementary Fig. 1 | Cell type marker gene expression in cell clusters** Violin plots of log-normalized gene expression (y-axis, right hand side) for cell type markers (y-axis, left hand side) used to annotate cell clusters (x-axis) for known cell types. The colors correlate to clusters from Figure 1.d.


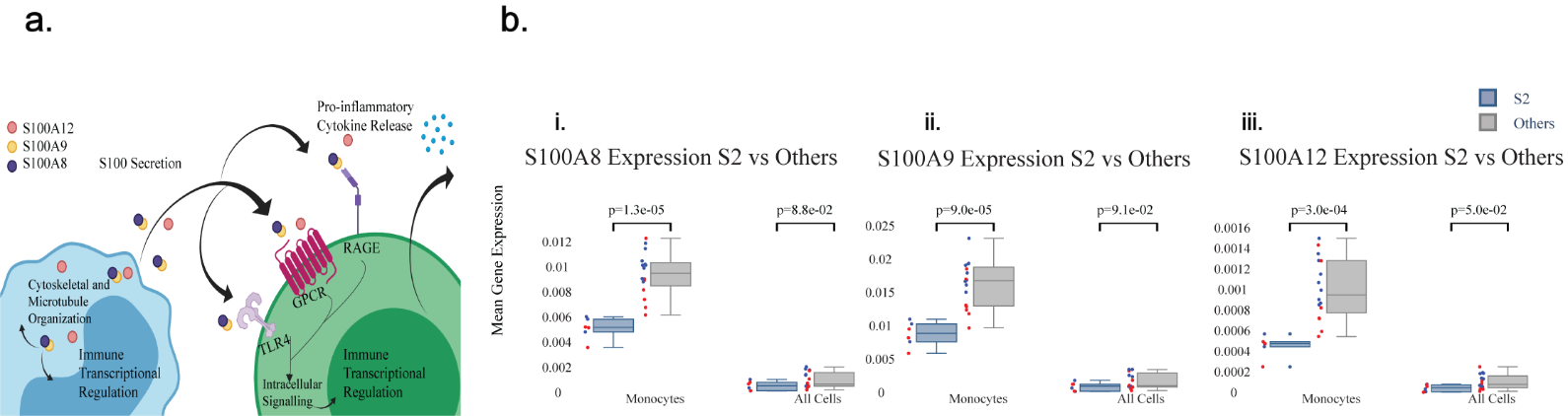


**Supplementary Fig. 2 | S100 pathway exhibits individual-specific regulation (a)** Simple schematic illustrating the role of S100A8, S100A9, and S100A12 genes in immune regulation. **(b)** Normalized mean gene expression of S100A8, S100A9, and S100A12 genes for S2 showing significant downregulation in monocytes as compared to all cells.


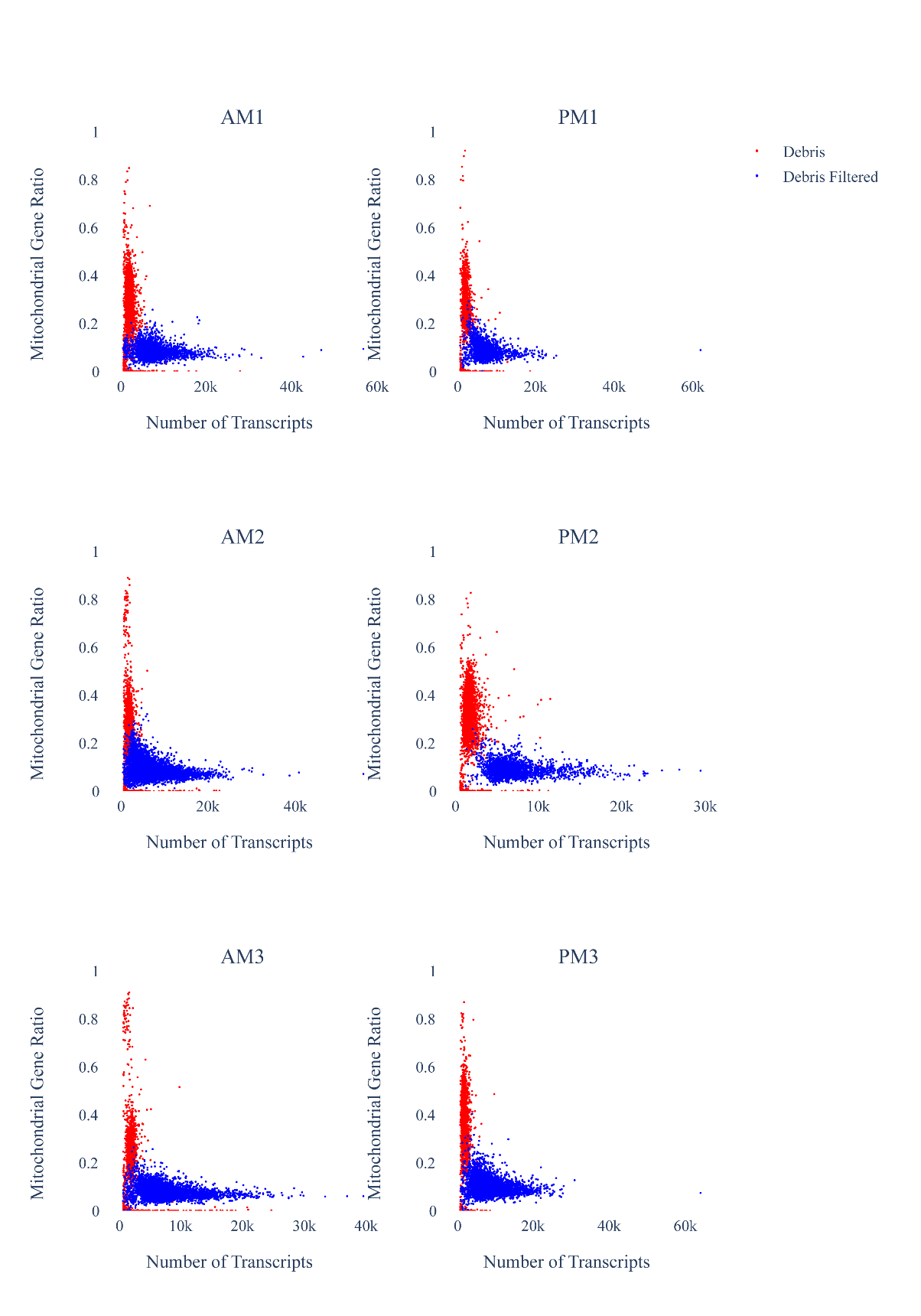


**Supplementary Fig. 3 | Characterization of debris removal pipeline across each time sample.** Scatter plots of the total number of transcripts (UMIs) detected for each barcode (x-axis), and the ratio of transcripts that are mitochondrial (y-axis). These barcodes are the union of barcodes called by 10X Cellranger and our debris filtering pipeline. Barcodes colored red were flagged as debris and removed. The debris filtering pipeline appears to detect barcodes that have both a low transcript count, and a high mitochondrial gene ratio, or a rare number of cells that appear to have 0 mitochondrial genes. The counts of barcodes removed for each sample are in Table S6.


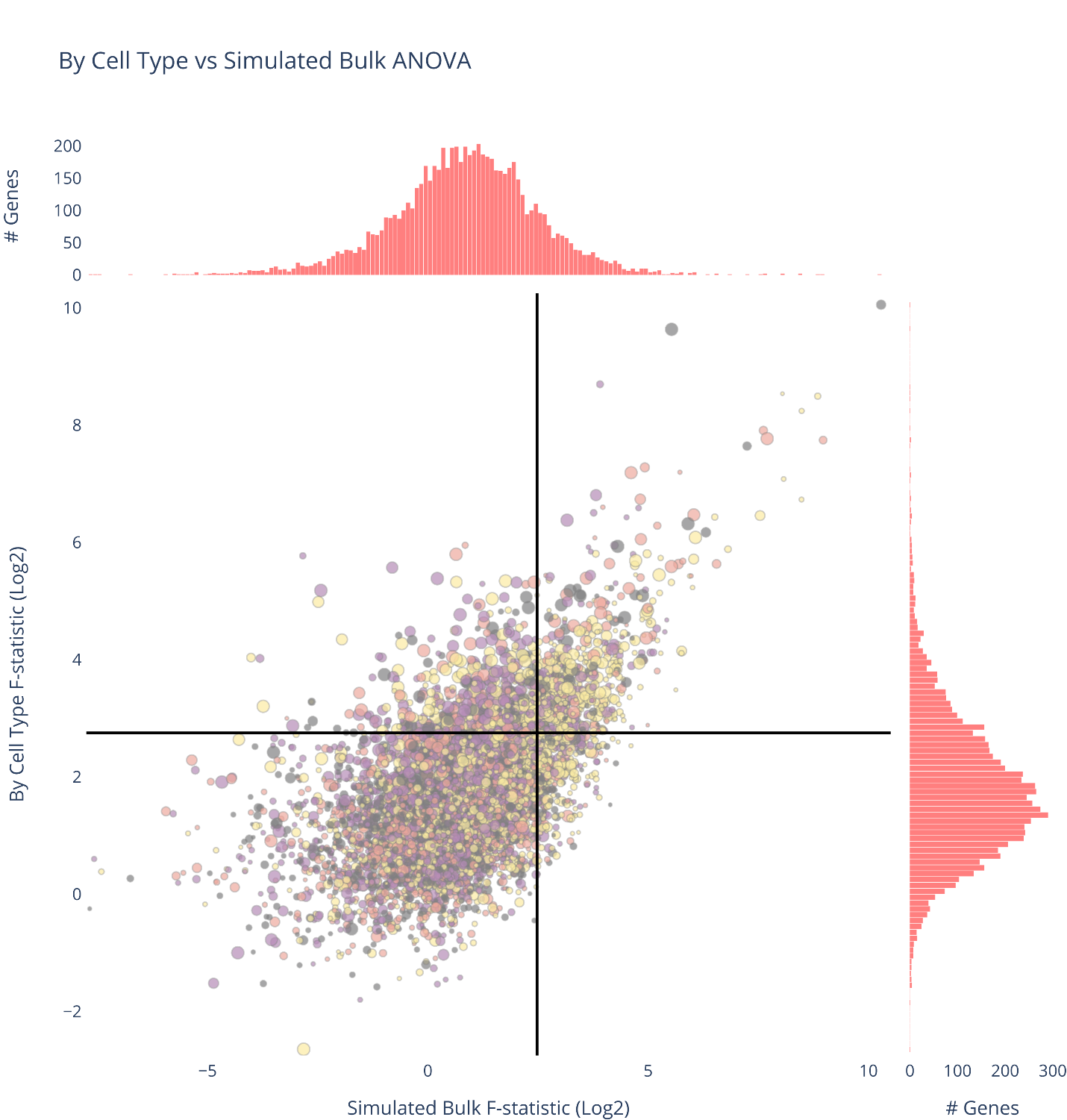


**Supplementary Fig. 4 | Comparison of individual specificity by cell type vs in simulated bulk data.** Magnitude (log_2_ F statistic) of the variability in expression of genes between subjects, accounting for each cell type separately (y) and in simulated bulk (x). 1284/7034 (18.3%) of genes are above the subject specificity significance line (FDR < 0.05, multiple comparison corrected) and are classified as subject-specific. Of these, only 637/1284 (49.6%) are also detected as subject-specific when simulating bulk RNA reads, despite the significantly lower multiple comparison correction burden (7034 tests as compared to 28,136 tests in the cell type case).


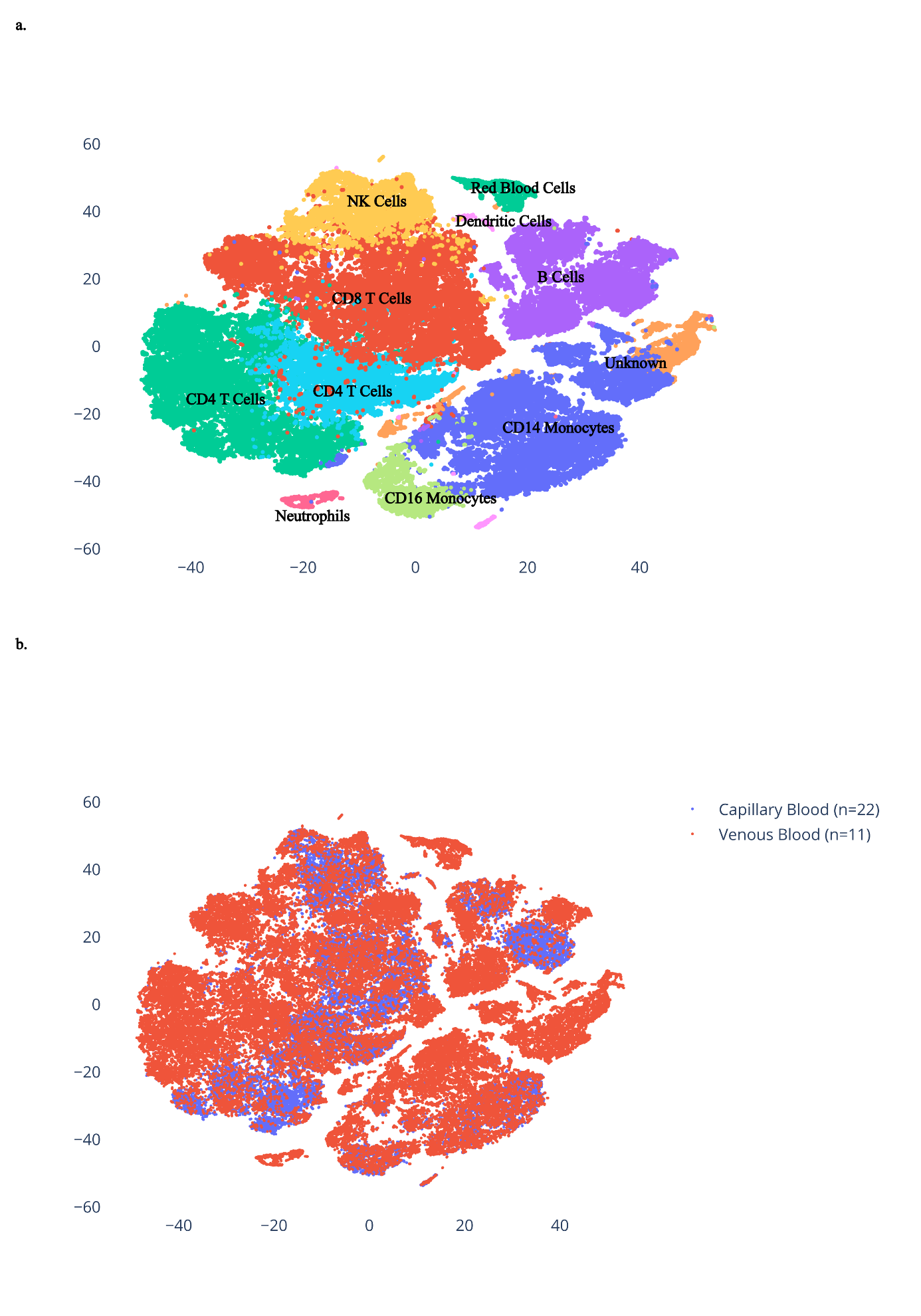


**Supplemental Fig. 5 | Merged projection of capillary and venous blood cells.** Capillary blood cells from this study (n=22) and venous blood cells from 3 other studies (n=11) were projected into a joint latent space using scVI. (a) Agglomerative clustering with n=13 clusters was performed to identify cell types, and annotated using known cell type markers (b) Capillary blood cells cluster together with venous blood cells, with the exception of one cluster of B cells unique to capillary cells, as well as 3 cell types unique to the venous blood sample: red blood cells, dendritic cells, and neutrophils, which are likely filtered out via laboratory procedures and the computational debris filtering pipeline.


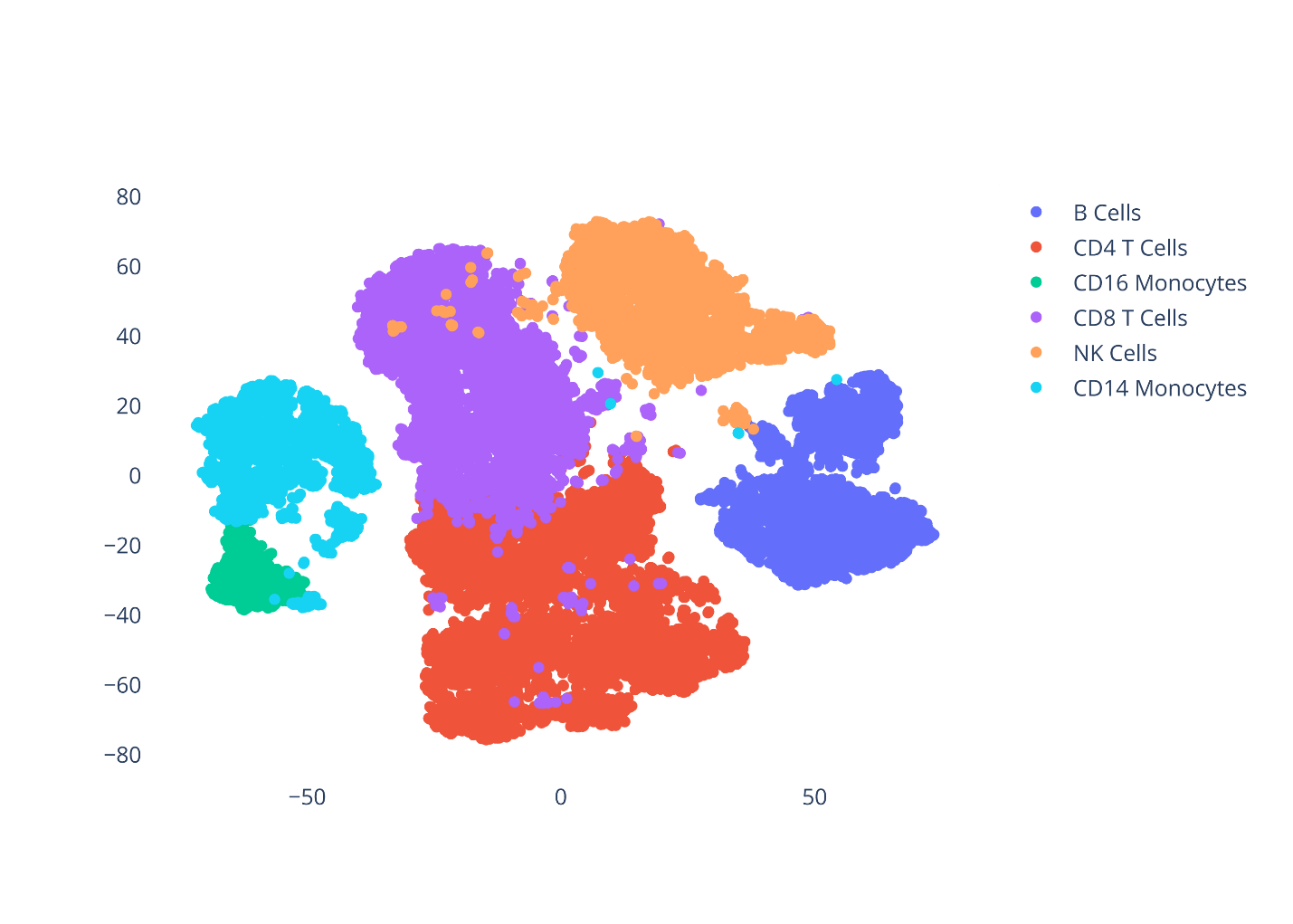


**Supplemental Fig. 6 | Immune cell type clusters detected in capillary blood.** 2-dimensional t-SNE projection of the transcriptomes of all cells in all samples obtained from agglomerative clustering of latent gene expression. Cell clusters were annotated and grouped based on the markers presented in Table S2. Small unidentifiable clusters were are not included in the figure.

**Table S1:** Genes that ranked in top 20 that had pre-existing literature tying to circadian/diurnal expression

| **Gene** | **DOI Reference** |
| --- | --- |
| DDIT4 | 10.7554/eLife.20214.001, 10.1073/pnas.1800314115 |
| SMAP2 | 10.1038/s41398-019-0671-7 |
| RPL19 | 10.1128/MCB.00701-15 |
| RPS9 | 10.1073/pnas.1515308112 |
| PCPB1 | 10.1038/s41556-019-0441-z |
| RPS2 | 10.1073/pnas.1601895113 |
| RBM3 | 10.1038/srep02054 |
| COX5B | 10.1152/physiolgenomics.00066.2007 |

**Table S2:** Marker genes used to annotate clusters with specified cell population identity.

| **Cells** | **Marker Genes** |
| --- | --- |
| CD14 Monocytes | CD14, LYZ |
| CD16 Monocytes | FCGR3A, MS4A7 |
| CD4 T Cells | IL7R,CCR7 |
| CD8 T Cells | KLRG1, CD8A, CD8B |
| Natural Killer (NK) Cells | GNLY, KLRF1, KLRD1 |
| B Cells | BANK1, CD79A, CD79B, CD19 |

**Table S3:** Subject age and demographics. All subjects indicated to be healthy during the study.

| **Subject** | **Age** | **Gender** |
| --- | --- | --- |
| S1 | 32 | M |
| S2 | 41 | M |
| S3 | 34 | F |
| S4 | 26 | F |

**Table S4:** Details of studies used to get healthy venous blood single-cell RNA sequencing dataset for comparison with capillary blood.

| **Subject** | **Age** | **Gender** | **Corresponding DOI** | **Corresponding Study Identification** |
| --- | --- | --- | --- | --- |
| S1 | 21 | M | <https://doi.org/10.1038/s41598-020-59827-1> | Pre-THC-S1 |
| S2 | 21 | M | <https://doi.org/10.1038/s41598-020-59827-1> | Pre-THC-S2 |
| S3 | 63 | F | <https://doi.org/10.1126/sciimmunol.abd1554> | Sample 5_Normal 1 scRNA-seq [SW107] |
| S4 | 54 | F | <https://doi.org/10.1126/sciimmunol.abd1554> | Sample 13_Normal 2 scRNA-seq [SW115] |
| S5 | 67 | F | <https://doi.org/10.1126/sciimmunol.abd1554> | Sample 14_Normal 3 scRNA-seq [SW116] |
| S6 | 63 | M | <https://doi.org/10.1126/sciimmunol.abd1554> | Sample 19_Normal 4 scRNA-seq [SW121] |
| S7 | 50 | M | <https://doi.org/10.1073/pnas.1907883116> | CT1 |
| S8 | 70 | F | <https://doi.org/10.1073/pnas.1907883116> | CT2 |
| S9 | 60 | F | <https://doi.org/10.1073/pnas.1907883116> | CT3 |
| S10 | 70 | F | <https://doi.org/10.1073/pnas.1907883116> | CT4 |
| S11 | 80 | M | <https://doi.org/10.1073/pnas.1907883116> | CT5 |

**Table S5:** Number of genes in different cell types that is specific to each subject.

|  | B Cells | Monocytes | NK Cells | T Cells | Any |
| --- | --- | --- | --- | --- | --- |
| S1 | 55 | 67 | 58 | 269 | 400 |
| S2 | 24 | 94 | 49 | 58 | 190 |
| S3 | 55 | 149 | 70 | 150 | 353 |
| S4 | 49 | 36 | 34 | 44 | 131 |

**Table S6:** Statistics for debris removal pipeline.

|  | Cellranger Called | Removed | Added | Final # Cells | % Removed |
| --- | --- | --- | --- | --- | --- |
| AM1 | 5808 | 2662 | 21 | 3167 | 45.83 |
| PM1 | 3144 | 1302 | 12 | 1854 | 41.41 |
| AM2 | 8772 | 2037 | 20 | 6755 | 23.22 |
| PM2 | 6172 | 3587 | 0 | 2585 | 58.12 |
| AM3 | 6684 | 1408 | 10 | 5286 | 21.07 |
| PM3 | 7974 | 2370 | 4 | 5608 | 29.72 |

|  | **Description** | **File Name** |
| --- | --- | --- |
| **Table** **S7** | Differential expression analysis for each cluster and cell type of the combined capillary blood (n=22) dataset | cluster_differential_expression.xlsx |
| **Table S8** | Differential expression analysis for all clusters between capillary blood (n=22, this study), and venous blood (n=11, external studies) | capillary_vs_venous_differential_expression.xlsx |
